# Patient-specific risk profile associated with early-onset primary osteoarthritis of the shoulder: is it really primary?

**DOI:** 10.1007/s00402-021-04125-2

**Published:** 2021-08-18

**Authors:** Fabian Plachel, Doruk Akgün, Jan-Philipp Imiolczyk, Marvin Minkus, Philipp Moroder

**Affiliations:** Center for Musculoskeletal Surgery, Charité—Universitaetsmedizin Berlin, Campus Virchow, Augustenburger Platz 1, 13353 Berlin, Germany

**Keywords:** Primary glenohumeral osteoarthritis, Risk profile, Young population, Eccentric osteoarthritis

## Abstract

**Introduction:**

Although age is considered to be the major risk factor of primary glenohumeral osteoarthritis (GOA), younger population may suffer from degenerative changes of the shoulder joint without evidence of any leading cause. The purpose of this study was to investigate the risk profile in young patients suffering from presumably primary GOA.

**Methods:**

A consecutive group of 47 patients undergoing primary shoulder arthroplasty for early-onset GOA below the age of 60 years at time of surgery was retrospectively identified and prospectively evaluated. Patients with identifiable cause for GOA (secondary GOA) were excluded. The resulting 32 patients (mean age 52 ± 7 years; 17 male, 15 female) with primary GOA were matched by age (± 3 years) and gender to 32 healthy controls (mean age 53 ± 7 years; 17 male, 15 female). Demographic data and patient-related risk factors were assessed and compared among both groups to identify extrinsic risk factors for primary GOA. Patients were further subdivided into a group with concentric GOA (group A) and a group with eccentric GOA (group B) to perform a subgroup analysis.

**Results:**

Patients had a significantly higher BMI (*p* = 0.017), were more likely to be smokers (*p* < 0.001) and to have systematic diseases such as hypertension (*p* = 0.007) and polyarthritis (*p* < 0.001) and a higher Shoulder Activity Level (SAL) (*p* < 0.001) when compared to healthy controls. Furthermore, group B had a significantly higher SAL not only compared to healthy controls but also to group A, including activities such as combat sport (*p* = 0.048) and weightlifting (*p* = 0.01).

**Conclusions:**

Several patient-specific risk factors are associated with primary GOA in the young population, as well as highly shoulder demanding activities in the development of eccentric GOA. Consequently, a subset of young patients with eccentric primary GOA could in reality be secondary due to a muscular imbalance between internal and external rotators caused by improper weight training.

**Level of evidence:**

III, Case–Control study

**Supplementary Information:**

The online version contains supplementary material available at 10.1007/s00402-021-04125-2.

## Introduction

Glenohumeral osteoarthritis (GOA) is a well-described pathology reported mostly in the elderly [1–3]. Although the pathoetiology is multifactorial, age is considered to be the major risk factor [4]. Nevertheless, also the younger population suffers from degenerative changes of the shoulder joint [5]. Predisposing exogenous factors, such as inflammation or trauma [6], have been attributed to degradation and loss of articular cartilage. Consequently, GOA has been divided into primary (unspecific) and secondary (specific) forms depending on whether or not there is an identifiable underlying cause. While the latter is by far more common among the younger patients, the prevalence of primary GOA ranges from 2 to 10% in the 40–55 years age group [2], however, significantly increasing during the last decade [7]. Risk factors for primary GOA have been poorly studied. While older age and concomitant knee osteoarthritis as well as the critical shoulder angle are determining risk factors for primary GOA in elderly [3, 8], static posterior subluxation of the humeral head (PSHH) before the development of posterior bone erosion of the glenoid is identified as a risk factor associated with primary GOA in young patients [9, 10].

Therefore, the purpose of this study was to investigate the risk profile in young patients suffering from presumably primary GOA. It was hypothesized that (1) currently unrecognized patient-specific exogenous risk factors predispose primary GOA and (2) patients with static PSHH differ from those with centered humeral head in terms of their risk profile.

## Materials and methods

### Study participants:

**Fig. 1:**
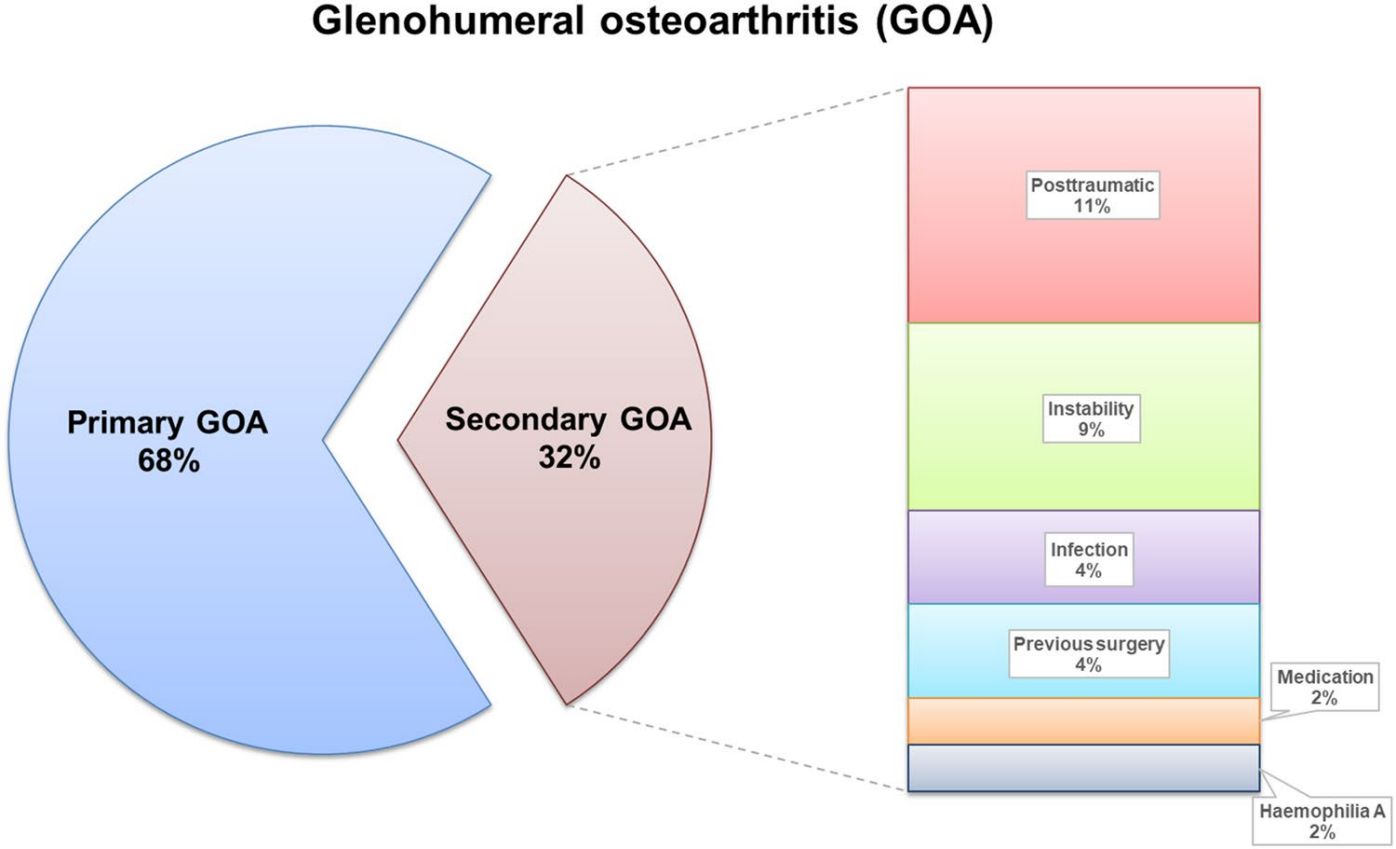
Pie chart showing an overview of primary and secondary glenohumeral osteoarthritis (GOA) among the study population (*n* = 32). If an open or arthroscopic stabilization surgery or rotator cuff repair was performed (i.e. term “previous surgery”), patients were excluded

Patients below the age of 60 years at the time of surgery, who underwent primary shoulder arthroplasty from 2010 to 2016 for GOA, were identified in the database of our institution. A total of 47 patients were extracted. Based on the pathoetiology, 15 patients with secondary GOA (e.g. previous macrotrauma, open/arthroscopic stabilization surgery or rotator cuff repair) were excluded from the study (Fig. 1). All patients had a symptomatic GOA in terms of severe pain and limited range of motion.

Those included were contacted by telephone and invited to complete a questionnaire, which has been developed based on the results of a literature review. It collected data on (1) patient demographics, (2) operation type and (3) patient-related factors. Details of the patient’s indication for surgery were verified using hospital medical notes. Furthermore, age- (± 3 years) and sex-matched healthy controls without any history of shoulder complaints were prospectively recruited among non-health-care receiving visitors to our hospital to conduct a 1:1 matching. Those who accepted the invitation received a modified version of the questionnaire including Sect. 1 and 3 mentioned above. Data collection was conducted in the period from November to December 2018. Ethical committee approval and written informed consent from all participants were obtained.

### Risk factor assessment

Patient characteristics at the time of surgery including weight, height, handedness, smoking habits, alcohol abuse, systematic diseases, and medications were extracted from the hospital records, whereas, the questionnaire was used to further assess participant characteristics. Any further exogenous environmental factors, such as previous injuries or infections, therapies (e.g. infiltration, physical therapy) or surgeries on any joint were collected. Both previous and current occupation as well as sport activities were documented, along with the shoulder activity level (SAL[11]).

The body mass index (BMI; body weight in kilograms/height in metres^2^) was computed, smoking was summarized in package-years (1 package-year = smoking 20 cigarettes/day for 1 year) and alcohol consumption in weekly units (U) of alcohol (1 U = 10 ml of pure alcohol). Cumulative calculation of life doses was determined for specified practiced activities (years/life). (Appendix 1).

### Patient subanalysis

Among all patients, preoperative radiographic images (axial images of computed tomography scan and conventional x-rays in true a.p., axial and Y-view) of the affected shoulder were screened and classified according to the modified Walch-system [12] to assess glenoid morphology. The analyses were performed by two independent raters (FP, PM). In cases with divergent results, the raters analyzed and discussed all images to reach consensus on one final rating. Accordingly, patients were subdivided in concentric primary GOA (group A; including type A glenoids) and eccentric primary GOA (group B; including both type B and C glenoids).

### Statistics

Statistical analyses were performed with IBM SPSS Statistics 24.0 software (IBM, Armonk, NY, USA) with the p-values being 2-tailed and the alpha level set to 0.05. Descriptive statistics, including frequencies of individual values as well as means, standard deviation, minimum and maximum values of continuous variables were calculated. Frequency tables and either the McNemar’s test or the Fisher’s exact test were used to find differences of dichotomic variables between two or more groups. The Kruskal–Wallis test was used to compare specific exogenous factors between more than two groups. After testing for normal distribution, matched-pair analyses (patients and healthy controls) were accomplished using Wilcoxon Signed Rank Test for ordinal or non-normally distributed continuous data, or paired t-test for continuous normally distributed variables. Furthermore, to analyze statistical differences between two specific groups, the Mann–Whitney *U* test for non-normally distributed data or independent *t*-test for normally distributed data were conducted.

## Results

A total of 32 patients (15 women) and 32 controls (15 women) were examined. Characteristics of both patients and healthy controls are summarized in Table 1. Overall, the BMI of patients was significantly higher (*p* = 0.017) compared to healthy controls. Furthermore, patients were also more likely to be current smokers (*p* < 0.001), to report systematic diseases such as hypertension (*p* = 0.007) as well as polyarthritis (*p* < 0.001) and a higher SAL (*p* < 0.001) with participation in combat sport (*p* = 0.020) and weightlifting (*p* = 0.011).

**Table 1:** Comparison of characteristics between patients and healthy controls

Variables	Patients *N* = 32	Controls *N* = 32	*p*-value
Age*, years	52 ± 7	53 ± 7	0.213
Sex, male:female	17:15	17:15	1.000
Height*, cm	174 ± 10	175 ± 13	0.569
Weight*, kg	84 ± 19	76 ± 15	0.058
Body mass index*, kg/m^2^	28 ± 5	25 ± 3	**0.017**
Smoking, %	69	26	**0.001**
Smoking*, package-years	12 ± 15	4 ± 10	**0.009**
Alcohol, %	70	67	0.670
Alcohol*, units	12 ± 17	15 ± 15	0.482
Systematic disease			
Diabetes mellitus, %	6	0	0.157
Epilepsy, %	3	0	0.317
Rheumatoid arthritis, %	3	3	1.000
Hypertension, %	31	3	**0.007**
Articular gout, %	3	3	1.000
Polyarthritis, %	44	3	**0.001**
Hypothyreosis, %	6	3	0.564
Cancer, %	3	0	0.233
Shoulder activity level*, points	1.9 ± 1.2	0.6 ± 0.7	**0.001**
Sports activity			
Combat sport, %	25	3	**0.020**
Life dose*, years/life	0.05 ± 0.03	0.01 ± 0.03	**0.012**
Gymnastics, %	13	3	0.180
Life dose*, years/life	0.01 ± 0.04	0.01 ± 0.05	0.595
Weight lifting, %	20	6	**0.011**
Bench press, max. weight*, kg	20 ± 50	1 ± 1	**0.035**
Boxing, %	6	0	0.157
Tennis, %	6	9	0.655
Ice hockey, %	6	0	0.122
Football, %	3	0	0.456
Diving, %	3	3	1.000
Previous shoulder surgery, %	28	0	**0.001**
Previous shoulder injection, %	44	3	**0.001**

^*^Data are reported as mean ± SD

Preoperative radiographics revealed a concentric GOA in 18 patients (56%), whereas, the humeral head was posteriorly decentered in 14 patients (44%). According to the modified Walch classification, a type A1 glenoid was found in 11 patients (34%), a type A2 in seven patients (22%), a type B1 in six patients (19%), a type B2 in five patients (16%), a type B3 in two patients (6%) and a type C in one patient (1%).

Subgroup analyses were performed to compare patient characteristics and patient-specific exogenous factors of patients (A and B) and healthy controls (Table 2). Similar to the overall comparison of patients and healthy controls, patients with a concentric GOA (group A) were more likely to be current smokers (*p* = 0.004), and suffer from systematic diseases such as hypertension (*p* = 0.002) as well as polyarthritis (*p* < 0.001) and had a higher SAL (*p* < 0.001) compared to healthy controls. Patients with an eccentric GOA (group B) had a significantly higher SAL compared to not only healthy controls but also patients with concentric GOA (group A), including activities such as combat sport (*p* = 0.048) and especially weightlifting (*p* = 0.01). Fittingly, a higher male to female ratio was identified in group B than group A (*p* = 0.016). While no differences regarding previous surgeries (*p* = 0.960) and injections (*p* = 0.191) were found between group A and B, patients in both group A and B were more likely to have previous surgeries (*p* < 0.001) and injections (*p* = 0.009) compared to healthy controls. Furthermore, both shoulders were affected by eccentric GOA in 43% of patients in group B compared to only 6% bilateral involvement in group A (*p* = 0.027).

**Table 2:** Comparison of characteristics between patients with a concentric primary glenohumeral osteoarthritis (GOA) (A), those with an eccentric primary GOA (B) and healthy controls

Variables	A *N* = 18	B *N* = 14	Controls *N* = 32	*p-value* Overall	*p-value* A vs. B	*p-value* A vs. Controls	*p-value* B vs. Controls
Age*, years	53 ± 7	52 ± 6	53 ± 7	0.969	0.848	0.979	0.801
Sex, male:female	6:12	11:3	17:15	**0.016**	**0.016**	0.241	**0.044**
Height*, cm	170 ± 9	178 ± 10	175 ± 13	0.134	**0.026**	0.125	0.550
Weight*, kg	78 ± 18	91 ± 17	76 ± 15	**0.023**	**0.046**	0.789	**0.006**
Body mass index*, kg/m^2^	27 ± 5	29 ± 5	25 ± 3	**0.011**	0.248	0.085	**0.002**
Bilateral glenohumeral osteoarthritis, %	6	43	n.a	n.e	**0.027**	n.e	n.e
Smoking, %	61	79	26	**0.001**	0.446	**0.004**	**0.001**
Smoking*, package-years	12 ± 18	11 ± 12	4 ± 10	**0.015**	0.869	**0.012**	**0.005**
Alcohol, %	78	64	67	0.665	0.453	0.556	0.493
Alcohol*, units	10 ± 11	14 ± 22	15 ± 15	0.550	0.421	0.235	0.994
**Systematic disease**							
Diabetes mellitus, %	0	14	0	0.088	0.183	1.000	**0.029**
Epilepsy, %	0	7	0	0.163	0.437	1.000	0.304
Rheumatoid arthritis, %	5	0	3	0.669	0.562	0.595	0.696
Hypertension, %	39	21	3	**0.005**	0.446	**0.002**	**0.043**
Articular gout, %	0	7	3	0.515	0.437	0.640	0.521
Polyarthritis, %	50	36	3	**0.001**	0.490	**0.001**	**0.007**
Hypothyreosis, %	6	7	3	0.821	0.692	0.595	0.521
Cancer, %	6	0	0	0.273	0.562	0.360	1.000
Shoulder activity level*, points	1.5 ± 1.0	2.5 ± 1.2	0.6 ± 0.7	**0.001**	**0.024**	**0.001**	**0.001**
Sports activity							
Combat sport, %	17	36	3	**0.013**	**0.048**	0.127	**0.007**
Life dose*, years/life	0.02 ± 0.06	0.06 ± 0.10	0.01 ± 0.03	**0.032**	0.100	0.219	**0.009**
Gymnastics, %	22	0	3	**0.025**	**0.049**	**0.050**	1.000
Life dose*, years/life	0.03 ± 0.06	0.00 ± 0.00	0.01 ± 0.05	0.231	0.086	0.240	0.514
Weight lifting, %	6	36	6	**0.011**	**0.010**	1.000	**0.003**
Bench press, max. weight*, kg	9 ± 41	32 ± 54	1 ± 1	**0.015**	**0.044**	0.188	**0.001**
Boxing, %	0	14	0	**0.025**	0.098	1.000	**0.029**
Tennis, %	6	7	9	0.316	0.692	0.398	0.413
Ice hockey, %	6	7	0	0.345	0.854	0.360	0.304
Football, %	6	0	0	0.273	0.370	0.360	1.000
Diving, %	6	7	3	0.359	0.356	0.674	0.254
Previous shoulder surgery, %	33	21	0	**0.033**	0.960	**0.001**	**0.010**
Previous shoulder injection, %	50	29	3	**0.029**	0.191	**0.001**	**0.009**

^*^Data are reported as mean ± SD; n.a. = not available; n.e. = not evaluated

## Discussion

Primary GOA in young patients remains a significant clinical challenge. Although treatment modalities are well studied, evidence analyzing the etiology of primary GOA is scarce. Identification of the underlying cause behind arthritic changes may affect the treatment algorithm and the patient’s prognosis. This is the first study in literature analyzing patient-specific exogenous risk factors predisposing to presumably primary GOA in the younger population.

Oh et al. published a large cohort series evaluating the prevalence and risk factors of GOA in the general population aged older than 65 years [3]. Their data demonstrated that older age and concomitant knee osteoarthritis were determining risk factors for GOA. Furthermore, GOA showed no gender predominance or association with obesity and other risk factors such as diabetes mellitus, hypertension and smoking in their cohort [3]. In contrast, a recent study investigating patients with GOA showed increased adipokine levels that correlated with BMI, suggesting that obesity may indeed affect non-weight bearing joints due to systemic metabolic stress [13]. Consistent with these results, the BMI of patients in our cohort was significantly higher compared to healthy controls. Furthermore, patients were also more likely to be current smokers, and suffer from hypertension as well as polyarthritis.

**Fig. 2:**
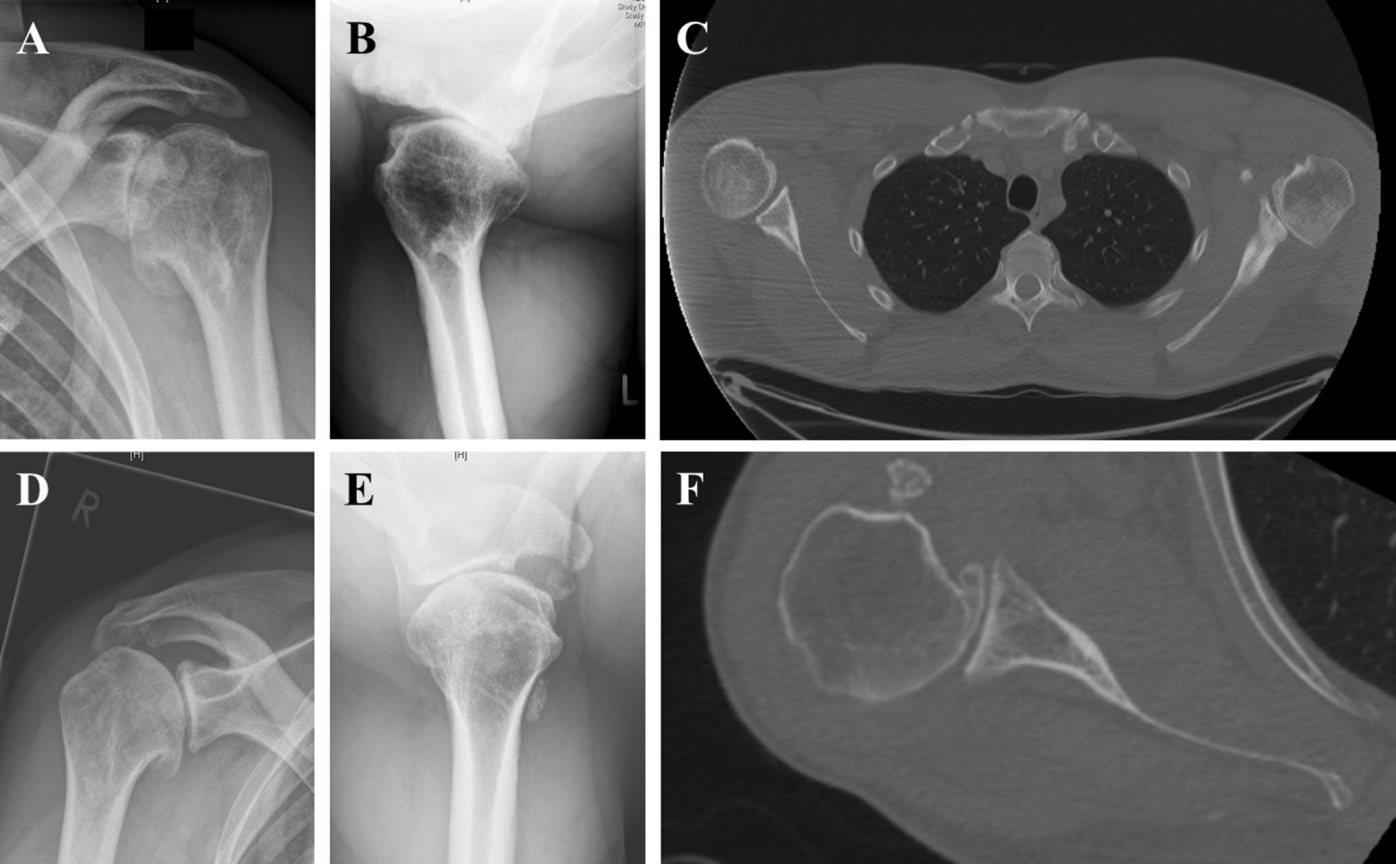
Preoperative radiographics of a 40-year-old male patient. The BMI is 29, he is a current smoker and the shoulder-activity level is 4 with participating in boxing, combat sport and weightlifting. He used to be a professional bodybuilder. **a**–**c** Conventional x-rays with a.p. **a** and axial **b** images revealing a severe primary glenohumeral osteoarthritis (GOA) of the left shoulder. Computed tomography scan showed a posterior subluxation of the humeral head with posterior glenoid erosion. **d**–**f** Three years later, the patient presented with progressive shoulder complaints on the contralateral shoulder. Radiographs revealed a similar posterior eccentric GOA of the right shoulder joint

**Fig. 3:**
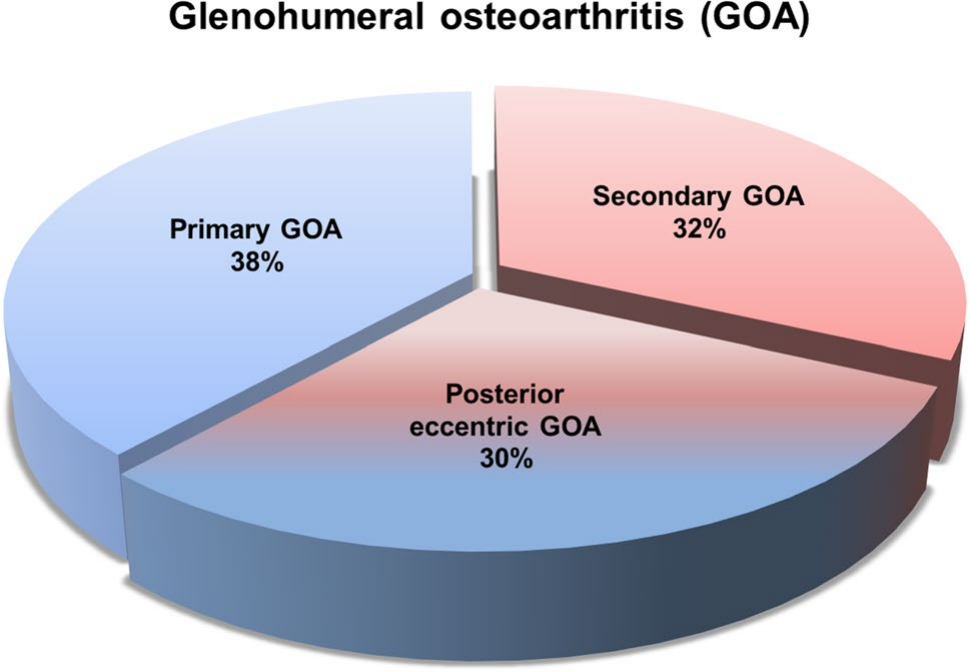
Pie chart demonstrating our findings with regard to the pathogenesis of glenohumeral osteoarthritis (GOA) in a young population (*n* = 32)

The most interesting finding of this study was the significantly higher shoulder demanding activity level in patients with an eccentric GOA compared with both healthy controls and patients with concentric GOA. The pathoetiology of PSHH with posterior degenerative changes of the glenoid remains unclear [9]. Walch et al. described PSHH in young patients in the context of primary GOA as a pre-osteoarthritic deformity, with subluxation of the humeral head preceding posterior glenoid erosion [5] (Fig. 2). That earliest form of the osteoarthritic evolution was named ‘B0’ glenoid [9]. However, it is still unclear whether PSHH predisposes glenoid retroversion due to eccentric joint mechanics or vice versa. Causes for PSHH appear to be multifactorial and are likely dependent on an interaction of bony and soft-tissue factors including muscle balance. The ABC classification distinguishes between constitutional (C1) and acquired static (C2) posterior shoulder instability [14]. The theoretical pathomechanism for C1 comprises muscular imbalances leading to progressive posterior shoulder joint decentering and eccentric wear. This interaction becomes clearer when analyzing the shoulder deformity in patients with brachial plexus palsy (BPBP). The occurrence of BPBP leads to muscular imbalance around the shoulder joint with internal rotation contracture with consequent asymmetric growth of the glenoid with excessive glenoid retroversion and early posterior subluxation of the humeral head [15]. Muscular imbalance developed later in life may also cause a dynamic posterior humeral head subluxation leading to secondary posterior bone erosion of the glenoid and eccentric GOA in the young population. Maintaining the appropriate agonist–antagonist strength ratios is essential in providing joint stability [16]. However, the demands of certain sport activities may alter the natural strength ratios. It has been shown that weight training routines often focus on the selection of large muscle groups such as pectoralis major, upper trapezius and deltoids, subsequently neglecting muscles responsible for shoulder stabilization, such as rotator cuff and scapular musculature [17]. Recent studies were able to find greater strength in the abductor and internal rotator musculature among weight training participants compared to controls, however, the shoulder external rotators and lower trapezius musculature were not significantly stronger [17, 18]. Thus, this creates an imbalance of a muscle force couple which may alter the normal shoulder function and lead subsequently to primary GOA with posterior glenoid wear in young patients [19]. Given the fact, that shoulder demanding activity levels with participation in combat sport and weightlifting was most common in patients with a posteriorly decentering GOA and comparable between healthy controls and patients with concentric GOA, the muscular imbalance may play an important role especially in the eccentric GOA. Consequently, a subset of young patients with eccentric primary GOA could in reality be secondary due to a muscular imbalance between internal and external rotators caused by improper weight training (Fig. 3).

The significantly higher number of male patients and higher weight and height in the group B compared to the group A can be explained by the fact, that weightlifting and combat sports are more commonly performed by males than females. Furthermore, 33% of the females in group B also reported performing weightlifting routinely, while none of the female patients did so in group A. Thus, male gender cannot be postulated as an independent risk factor in developing a posterior decentering GOA. Furthermore, some patients had undergone either a previous arthroscopic surgery other than stabilization surgery and rotator cuff repair or injection of the affected shoulder joint. However, the dynamic and static stabilizers of the glenohumeral joint were found to be macroscopically intact in all cases. Moreover, the shoulder injection was performed within the last 12 months prior to primary shoulder arthroplasty and thus not directly associated to the development of GOA.

In patients with an early-onset primary GOA, the causes leading to this pathology can be too subtle to be recognized. Nonetheless, it is likely that factors other than age are leading to the development of primary GOA making it indeed a secondary GOA. This difference might be of major importance at least for a subset of patients with decentering GOA as it might affect the prevention and treatment strategies. A proper education of individuals pursuing weight training may prevent the development of decentering GOA in a subset of patients. In patients with a PSHH, a targeted physical therapy program with a balanced strengthening of the shoulder girdle may theoretically (1) balance strength ratios of force couples, (2) provide soft tissue mobility balance and (3) achieve a better centering of the humeral head [17]. In patients with a PSHH and failed physical therapy program without evidence of significant arthritic changes an arthrolysis of the anterior compartment and posterior capsular shift could improve soft tissue balancing. However Walch et al. were not able to correct arthrogenic posterior subluxation despite different arthroscopic treatment modalities [5]. In cases with excessive glenoid retroversion corrective osteotomy of the glenoid can be performed, however, the correction of glenoid retroversion does not necessarily correct the PSHH [20]. This can be due to significant differences of scapula morphology in terms of an increased anterior glenoid offset, which is not addressed with retroversion corrective osteotomy [21]. Surgical treatment of young patients with advanced osteoarthritis and B-type glenoids remains a challenge. In comparison with type-A glenoid, the anatomic total shoulder arthroplasty in B glenoids incur a higher failure rate due to glenoid loosening or recurrent posterior instability [22, 23]. Reverse shoulder arthroplasty may provide reliable improvement of shoulder function in these patients with B glenoids [24, 25], however, longevity is a major concern.

This study has some limitations. The major drawback of the study is the retrospective study design, which limits the reliability and completeness of our results. Furthermore, a quantitative assessment of the shoulder demanding activities through a questionnaire is difficult, so a threshold for shoulder demanding activities, which may lead to eccentric GOA could not be given. However, it should be noted that not necessarily the level of the performed shoulder activities is the possible cause of a GOA, but maybe rather a muscular imbalance due to improper training. Another limitation is the risk for underpowering for some of the analyzed risk factors in this study, despite portraying one of the largest series of young patients with primary GOA reported in literature. As our control group included healthy individual guests not receiving health care in order to avoid selection bias, potential osteoarthritic changes of the shoulder joint could not be ruled out definitely. Nevertheless, none of them complained any current or previous shoulder problems.

## Conclusion

Several patient-specific risk factors such as BMI, hypertension and polyarthritis are related to the pathogenesis of primary GOA. Furthermore, highly shoulder demanding activities lead to muscular imbalance and thus, may play an important role in the development of eccentric GOA. Consequently, a subset of young patients with eccentric primary GOA could in reality be secondary due to a muscular imbalance between internal and external rotators caused by improper weight training.

## Supplementary Information

Below is the link to the electronic supplementary material.

Supplementary file1 (PDF 258 kb)
