# Supplementary material for: Patient-specific risk profile associated with early-onset primary osteoarthritis of the shoulder: is it really primary?
Source: Arch Orthop Trauma Surg. 2021 Aug 18;143(2):699–706. doi: 10.1007/s00402-021-04125-2 (PMC9925503; doi:10.1007/s00402-021-04125-2)
Supplement: Supplementary file 1 — Supplementary file1 (PDF 258 kb) [file 402_2021_4125_MOESM1_ESM.pdf]

PART 1:

**Surname, Name:**			**Date of birth:**	
**Sex:**	O M *(1)*	O F *(2)*	**Height:**	**Weight:**

Handedness: O left *(1)* O right *(2)*

PART 2:

**Diagnosis:**	O primary GOA *(1)*	O secondary GOA *(2)*	
**Surgery:**	O HSA *(1)*	O TSA *(2)*	O RTSA *(3)*
**Side:**	O left *(1)*	O right *(2)*	
**Date**	_ _ _ _ _ _	*(dd.mm.yyyy)*	

PART 3:

**Current profession:**	_ _ _ _ _ _ _ _ _ _ _ _ _ _	O >50% oH	O <50% oH	***oH = over Head***
**Previous profession:**	1)	O >50% oH	O <50% oH	
	2)	O >50% oH	O <50% oH	
	3)	O >50% oH	O <50% oH	
**Smoking:**	O Yes *(1)*	**since:** _ _ _ _ (yyyy)		**amount:** _ _ _ _
	O Never *(2)*	**Pack-years:** _ _ _ _		
**Alcohol consumption:**	O Yes (1)	**since:** _ _ _ _ (yyyy)		**amount:** _ _ _ _

Pease estimate your weekly alcohol intake: _

_ _ _ **beers (50cl)**	_ _ _ **wine (20cll)**	_ _ _ **liqueur (2cl)**

	O No (2)	**Not anymore since:** _ _ _ _	

**Diabetes mellitus:**	O Yes *(1)*	**type:**	**therapy:** _ _ _ _ _ _ _ _ _ _ _ _ _
	O No *(2)*	**since:** _ _ _ _ (y)	_ _ _ _ _ _ _ _ _ _ _ _ _ _ _ _ _ _ _ _
**Epilepsy:**	O Yes (1)	**since:** _ _ _ _ (y)	**therapy:** _ _ _ _ _ _ _ _ _ _ _ _ _
	O No (2)	**number:** _ _ _ _	_ _ _ _ _ _ _ _ _ _ _ _ _ _ _ _ _ _ _ _
**Electricity accident**	O Yes *(1)*	**When?** _______	**How much voltage?** __________
	O No *(2)*		

Others

Pre-existing illnesses: O Yes *(1)* O No *(2)*

_ _

_ _

_ _

Regular medication: O Yes *(1)* O No *(2)*

_ _

_ _

_ _

1) **Have you ever received treatment (e.g. physiotherapy, surgery) for musculoskeletal disorders?**

**Physiotherapy:**	O Yes *(1)* O No *(2)*	
	O Which specific joint? ____________________	
	O When:	*(dd.mm.yyyy)*
	O How often? How many treatments?	
**Surgery:**	O Yes *(1)* O No *(2)*	
	O Which specific joint? ____________________:	
	O When:	*(dd.mm.yyyy)*
	O Which kind of suergy? _________________________	

**Infiltration:**	O Yes *(1)* O No *(2)*	
	O Which specific joint? ____________________	
	O When:	*(dd.mm.yyyy)*
	O How often in total? ___________	
	O Which substance / drug? ___________:	
**Cortisone-therapy:**	O Yes (1) O No *(2)*	
	O Which specific joint? ____________________	
	O When:	*(dd.mm.yyyy)*
	O Indication:	
	O How often? How many? _____________________	

O Which dosage? ______________________

2) Have you ever experienced high levels of radiation due to diagnostic or therapeutic (e.g. oncologic or nuclear medical) treatment?

O Yes *(1)* O No *(2)*

O When: *(dd.mm.yyyy)*

O Indication:

O What kind of treatment?

3) Have you ever experienced following events / illnesses?

**Trauma or accident**			
	O Yes *(1)* O No *(2)*	**When:**	*(dd.mm.yyyy)*
**Course of accident:**			
**Affected joint / bone:**			
**Diagnosis:**	1)		
	2)		
	3)		
**Surgery:**	O Yes *(1)* O No *(2)*	**Details:**	
**Immobilization:**	O Yes *(1)* O No *(2)*	**How long?**	

Infection (Inflammation)				
	O Yes *(1)*	O No *(2)*	**When:**	*(dd.mm.yyyy)*
**Localization**				
**Affected side**	O right	O left	O both	
**Bacteria:**				
**Hospitalization:**				
**Surgery:**	O Yes *(1)*	O No *(2)*	**Details:**	
**Antibiotics:**	O Yes *(1)*	O No *(2)*	**Which one?**	

4) **Have you ever performed one of the following sport / exercises for recreation or professionally? Please specify for how many years.**

O Ice hockey:	O Combat sport:
O Boxing:	O American Football:
O Gymnastics:	O Tennis:
O Fitness / Gym:	
O Weight lifting:	
O Bench press:<br><br>- *Maximum weight: _ _ _ kg*	O Diving:<br><br>- diving accident?<br><br>- Caisson disease?

5) **Please specify what other sports you have performed in your entire life. Please specify for how often and for how many years and at what level (e.g. recreation / professional).**

1)

2)

3)

4)

5)
